# Identification of *De Novo* Copy Number Variants Associated with Human Disorders of Sexual Development

**DOI:** 10.1371/journal.pone.0015392

**Published:** 2010-10-26

**Authors:** Mounia Tannour-Louet, Shuo Han, Sean T. Corbett, Jean-Francois Louet, Svetlana Yatsenko, Lindsay Meyers, Chad A. Shaw, Sung-Hae L. Kang, Sau Wai Cheung, Dolores J. Lamb

**Affiliations:** 1 Scott Department of Urology, Baylor College of Medicine, Houston, Texas, United States of America; 2 Department of Molecular and Cellular Biology, Baylor College of Medicine, Houston, Texas, United States of America; 3 Department of Molecular and Human Genetics, Baylor College of Medicine, Houston, Texas, United States of America; Health Canada, Canada

## Abstract

Disorders of sexual development (DSD), ranging in severity from genital abnormalities to complete sex reversal, are among the most common human birth defects with incidence rates reaching almost 3%. Although causative alterations in key genes controlling gonad development have been identified, the majority of DSD cases remain unexplained. To improve the diagnosis, we screened 116 children born with idiopathic DSD using a clinically validated array-based comparative genomic hybridization platform. 8951 controls without urogenital defects were used to compare with our cohort of affected patients. Clinically relevant imbalances were found in 21.5% of the analyzed patients. Most anomalies (74.2%) evaded detection by the routinely ordered karyotype and were scattered across the genome in gene-enriched subtelomeric loci. Among these defects, confirmed *de novo* duplication and deletion events were noted on 1p36.33, 9p24.3 and 19q12-q13.11 for ambiguous genitalia, 10p14 and Xq28 for cryptorchidism and 12p13 and 16p11.2 for hypospadias. These variants were significantly associated with genitourinary defects (*P* = 6.08×10^−12^). The causality of defects observed in 5p15.3, 9p24.3, 22q12.1 and Xq28 was supported by the presence of overlapping chromosomal rearrangements in several unrelated patients. In addition to known gonad determining genes including *SRY* and *DMRT1*, novel candidate genes such as *FGFR2*, *KANK1*, *ADCY2* and *ZEB2* were encompassed. The identification of risk germline rearrangements for urogenital birth defects may impact diagnosis and genetic counseling and contribute to the elucidation of the molecular mechanisms underlying the pathogenesis of human sexual development.

## Introduction

The acquisition of a sexual phenotype depends on critical embryonic steps, which initially commit the bipotential gonad to either a testis or an ovary and direct normal morphogenesis of external genitalia. Disruption of these developmental processes occurs frequently in humans as reflected by the high prevalence in newborns of disorders of sexual development (DSD) ranging in severity from genital abnormalities to complete sex reversal. Failure of testis descent or cryptorchidism is found in 2% of full-term males [1]. Hypospadias or defects in the growth and closure of the external genitalia affect nearly 1 in 125 live male births [2]. Genital phenotypes that are not clearly male or female are estimated to occur in about 1 of 2000 to 4500 babies [3]. Despite their incidence, the molecular basis underlying the pathology of congenital genitourinary (GU) defects is surprisingly poorly understood. Fetal exposure to environmental toxicants [4], [5], as well as point mutations in a small subset of genes (see for review [6], [7]) can affect human urogenital tract development, but these known causes do not account for all of the large number of GU birth defects. Interestingly, as referenced in the Online database of Mendelian Inheritance in Man (http://www.ncbi.nlm.nih.gov/sites/entrez?db=omim), a significant number of these urogenital inborn errors are associated with major congenital malformations or multiple minor anomalies, a trait that is highly suggestive of a causative chromosomal abnormality. However, routine cytogenetic methods had led to earlier reports of low rates of structural defects associated with disorders of sexual development [8], [9].

The finding that several common syndromes (including mental retardation, developmental delay and autism) are caused by specific submicroscopic chromosomal rearrangements, opened up new avenues for dissecting complex human phenotypes [10], [11]. The development of comparative genomic hybridization (CGH) into a microarray format allowed the identification and diagnosis of cryptic deletions or duplications of genomic regions that were once invisible using traditional cytogenetic methods, including karyotype analysis and fluorescence *in situ* hybridization (FISH). Several of these subtle rearrangements occur in regions flanked by low-copy repeats and likely result from non-allelic homologous recombination between different copies of these repeats during meiosis. Such submicroscopic imbalances lead to copy number changes of DNA segments and can influence gene expression levels by directly disrupting genes or regulatory sequences, creating fusion genes or altering gene dosage. These structural chromosomal defects can cause disease as occurs in the microdeletion and microduplication syndromes [12], [13], [14], [15], [16] or confer risk of complex disorders [17], [18].

Cryptic chromosomal rearrangements are involved in the etiology of human reproductive disorders since Y chromosome microdeletions are associated with human male infertility. Based on this, we tested the hypothesis that submicroscopic chromosomal alterations, too small to be detected by routine cytogenetic methods, may exist in patients with human disorders of sexual development. We studied probands presenting with hypospadias, cryptorchidism and ambiguous genitalia, the most common genital defects seen in pediatric urology clinics. We compared the resolution of clinical detection of such cryptic abnormalities by microarray-based chromosomal screening and by the routinely used karyotype. We further analyzed the contribution of these structural anomalies to the observed GU phenotypes by studying their association with the genital traits, as well as their inheritance and their recurrence. For the first time, findings revealed the presence of frequent microdeletions and microduplications in the genome of children born with urogenital disorders and established *de novo* germline rearrangements as significant risk factors for developmental defects of human urogenital tract.

## Methods

### Ethics Statement, Human Subjects and Sample Collection

This study was approved by the Institutional Review Board Committee at the Baylor College of Medicine, Houston TX. Probands affected with unexplained syndromic and non-syndromic congenital genitourinary disorders including hypospadias, cryptorchidism or ambiguous genitalia were enrolled through Texas Children's Hospital and Ben Taub General Hospital, Houston TX. Known causes of these birth defects such as anomalies in the synthesis of testosterone or adrenal steroid hormones or exogenous modifiers were ruled out after examination by pediatric urologists or neonatologists. Written informed consents were obtained for infant/child subjects and from their parents. Blood was collected from the children during surgery for correction of the GU defects. Parents provided saliva specimens. Based on the novel CMA findings, additional cases were then identified through an existing database from Kleberg Cytogenetics Laboratory (Baylor College of Medicine, Houston TX). These additional probands were referred patients, mostly presenting with external genital ambiguity with or without subclinical phenotypes. Clinical indications at the time of the referral were taken from crude clinical comments on laboratory requisitions.

### CGH based Microarray Analysis (CMA)

High molecular weight genomic DNA isolated from peripheral blood or saliva was submitted for chromosomal microarray analysis (CMA) to the Clinical Cytogenetics Laboratory at Baylor College of Medicine. CMA is a clinically validated targeted CGH array that covers over 150 distinct human clinically relevant chromosomal loci [19], [20]. Three different versions of CMA have been used depending on the time of sample submission. The versions 5 and 6 contain 3 to 10 BAC/PAC clones per genomic disorder specific locus and subtelomeric regions, with CMA V.5.0 consisting of 853 BAC clones and CMA V.6.1 consisting of 1475 BAC clones with inclusion of 1 clone per band at 650 cytogenetic banding resolution. The newer version Oligo V.6 uses 42,640 oligonucleotides of 60 base pairs with an average of 20 to 40 oligonucleotides corresponding to each CMA V.6.1 BAC clone genomic locus. Importantly, data acquired from the array platforms CMA V.6.1 and CMA Oligo V6 were shown to be qualitatively comparable, allowing for cross comparison analysis [21]. One unique DNA reference served as a control for CMA analysis and was from a pregnancy-proven fertile, gender-matched individual without any familial history of congenital genitourinary defects. CMA procedures and data analyses were performed as previously described [19], [20], [21].

All data are MIAME compliant and have been deposited in a MIAME compliant database.

### Interpretation of CNV significance

Clinically significant CNVs included detection of well-characterized deletion/duplication syndromes, deletion/duplication >3 Mb in size or cytogenetically visible, and *de novo* deletions or duplications <1 Mb. Imbalances that were not associated with well-characterized human syndromes were defined as “likely benign” when the variant was well documented to occur in the normal population on the basis of public databases (http://projects.tcag.ca/variation) or internal lab experience which includes analysis of about 16,000 individuals. In cases in which non-polymorphic defects were <1 Mb in size and parental samples were unavailable, variants were considered to be CNVs of uncertain clinical significance. Maternally inherited copy changes were included in this latter category as the rearrangements may be causative without necessarily translating into similar abnormal GU traits in the female genitourinary tract.

### CNV confirmation

FISH analysis was used to validate selected CMA findings >150 Kb in size, using the standard clinical cytogenetics laboratory protocol [22]. Briefly, BAC clone DNA probes were labeled directly with Spectrum Orange-dUTP or Spectrum Green-dUTP using a commercially available kit (Abbot Molecular/Vysis). At least 10 metaphase and/or 50 interphase cells were scored for each hybridization. A control probe, labeled in the opposite color, was included in the same hybridization in order to confirm that cells were diploid (ploidy control).

Quantitative TaqMan copy number variant (CNV) assays (Applied Biosystems) were used as an alternate secondary confirmation to FISH analysis. All reactions with TaqMan CNV assays were performed in triplicate using the FAM dye label-based assay for the target of interest and the VIC dye label-based TaqMan CNV RNaseP for the internal controls. The targets were custom designed in the areas where most significant changes in the probes were detected. QPCR was performed with 20 ng gDNA according to the manufacturer's protocol in an Applied Biosystems One Step Plus Real-Time PCR System using the default universal cycling conditions. Relative quantitation analysis was done to estimate copy number for each sample by using the Copy Caller Software V1.0 (Applied Biosystems).

### Concurrent G-banding Karyotype

Metaphase preparations from PHA-stimulated patient lymphocyte cultures followed a standard protocol to obtain chromosomes at ≥600–50-band level [23]. Briefly, after being cultured for ∼72 hours in RPMI 1640 with 20% fetal bovine serum, lymphocytes were synchronized by the addition of thymidine for 24 hours of culture, followed by the addition of ethidium bromide and colcemid for the last 45 minutes and 25 minutes of culture, respectively. The cells were treated for 20 minutes with 0.075 M KCl and were fixed in 3∶1 methanol–acetic acid prior to staining. The chromosomes were GTG-banded, and ≥20 chromosomal spreads were examined.

### Statistical Analysis

To analyze the frequency of *de novo* copy number changes in affected GU patients compared to unaffected GU individuals (non-GU controls), two-tailed Fisher's exact test was performed and statistical significance determined using SPSS software. Since (i) *de novo* events were only observed in GU patients run on CMA V.6.1 and CMA Oligo V6 (n = 90 out of the total of 116 analyzed GU children) and (ii) data acquired from the array platforms CMA V.6.1 and CMA Oligo V6 were shown to be qualitatively comparable [21], comparison of frequencies was done for cases and non-GU controls run only on CMA V.6.1 and CMA Oligo V6. *P* values were also determined for each of the spontaneous events to evaluate their association with the GU phenotype as compared to their specific occurrence in individuals without GU defects (n = 8951). Significance threshold was set at *P* = 5.0×10^−2^.

## Results

### Detection of Non-Polymorphic Imbalances Leading to Variations in Copy Number in the Genome of Children Born With Disorders of Sex Development

High molecular weight genomic DNA was isolated from peripheral blood of 116 children presenting with unexplained cases of disorders of sexual development ranging in severity from penile growth or testicular descent anomalies to genitalia ambiguity or complete sex reversal. Since the primary goal of this study was to improve the diagnosis of these urogenital defects and rapidly translate the findings to the clinic, DNA was analyzed using an established CGH microarray platform available for clinical diagnosis (chromosome microarray assay, CMA) [19], [20]. This targeted array specifically assesses relative copy number changes for over 150 human clinically relevant chromosomal loci. One unique DNA reference served as a control for CMA analysis and was from a pregnancy-proven fertile, gender-matched individual without any familial history of congenital genitourinary defects. Copy number variants (CNVs) were classified based on their clinical significance (see Methods). Basically, CNV pathogenicity depended on whether a given CNV overlapped with a known genomic syndrome that includes urogenital defects among the clinical features, or was present in a patient with similar phenotype, was not a copy number variant in healthy individuals, arose de novo (but not exclusively) and contained at least one gene.

**Figure 1:**
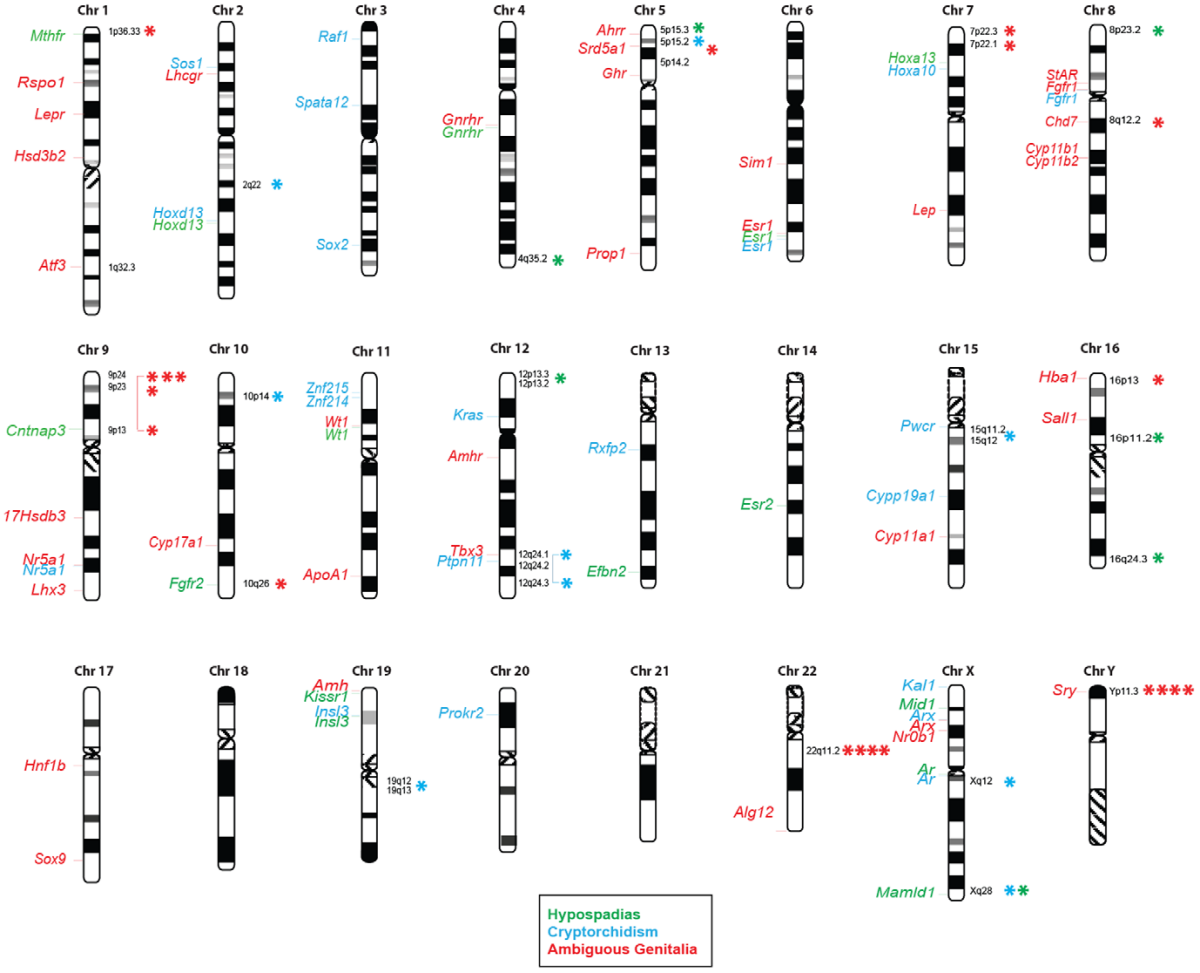
Comprehensive map of non-polymorphic copy number changes detected by CMA in patients with disorders of sex development. On the right, CMA detected imbalances were shown for each clinical condition (asterisks). To gain insight into the genomic distribution of the identified imbalances, all published single gene mutations associated with cryptorchidism (blue), hypospadias (green) and ambiguous genitalia (red) were reviewed and indicated on the left side of the chromosomes. References are available upon request.

Chromosomal imbalances were detected in 37 (31.9%) of the 116 patients analyzed (Table 1). When compared to the polymorphisms documented in public CNV databases (http://projects.tcag.ca/variation) or based on internal lab experience, which included analysis of about 16,000 individuals, 83.8% (31 of 37) of these defects were non-polymorphic (Table 1). FISH or qPCR secondarily validated these structural variants. They spanned the genome and affected sex chromosomes, as well as autosomal regions (Figure 1 and Tables 2–5), an observation consistent with the fact that male sexual development is governed by genes not restricted to the Y chromosome [7]. Most of the genomic rearrangements (25 of 37 *i.e.* 72.9%) were clinically significant copy number variants (Table 1 and Tables 2–4). Detection rates of these clinically relevant aberrations were slightly comparable between the three studied genital conditions (25% for ambiguous genitalia, 17.2% for hypospadias and 18.5% for cryptorchidism) (**Table 1**). Interestingly, these genomic abnormalities were noted in patients presenting with both syndromic, as well as non-syndromic genitourinary disorders (Table 1), with no statistically significant difference in their respective detection rates. From a clinical perspective, this latter observation stresses the importance of screening children presenting with isolated hypospadias or cryptorchidism, who usually are not referred for genetic testing.

**Table 1:** Submicroscopic imbalances revealed by CMA screening of children affected with syndromic and non-syndromic disorders of sex development.

	No Aberration	Chromosomal Aberrations			Total	Rate of Detection of Non-Poly morphic CNV (%)	Rate of Detection of Clinically Significant CNV (%)
	Normal	Benign CNV	Non-Polymorphic CNV				
			Clinically Significant	UCS			
**Ambiguous Genitalia**							
Isolated cases	21	3	11	1	36	33.3	27.8
Associated with other anomalies	18	1	4	1	24	20.8	16.0
Total	**39**	**4**	**15**	**2**	**60**	**28.3**	**25.0**
**Hypospadias**							
Isolated cases	12	0	3	2	17	29.4	17.7
Associated with other anomalies	8	1	2	1	12	25.0	16.7
Total	**20**	**1**	**5**	**3**	**29**	**27.6**	**17.2**
**Cryptorchidism**							
Isolated cases	15	0	2	0	17	11.8	11.8
Associated with other anomalies	5	1	3	1	10	40.0	30.0
Total	**20**	**1**	**5**	**1**	**27**	**22.2**	**18.5**
**Total**	**79**	**6**	**25**	**6**	**116**	**26.7**	**21.5**

Footnote: UCS: Uncertain Clinical Significance.

**Table 2:** List of pathogenic (P) copy number variations detected by CMA in patients presenting with gonadal dysgenesis or a referring diagnosis of ambiguous genitalia.

Locus	Case	GU defects	Associated anomalies	Concurrent G-banded karyotype	Array Platform	CNV(Signifi-cance)	Rearrangements seen in CMA	Min. size (Mb)	Secondary FISH	Min. genes	Inheritance
**1p36**	#1	Cloacal Exstrophy, Prominent Labioscrotal Folds, No Apparent Genital Tubercle	Midline Defect, imperforate Anus, Left Foot Anomaly	46, XX	CMA (Oligo V6.3)	Loss (P)	arr cgh 1p36.33(RP11-671C15->RP11-547D24)x1	1.25	ish del(1)(p36.33p36.33)(RP11-465B22-)dn	65	De Novo
**5p15**	#6	Ambiguous Genitalia (Referral)	None	46,XY,der(5)(qter->q34::p14->qter)	CMA (Oligo V6.2)	Loss (P)	arr cgh 5p15.33p14.2(RP11-487N22->RP11-91L13)x1, 16p13(RP11-349E19)x1	24	n/a	145	Unavailable
**9p23p24**	#12	Gonadal Dysgenesis	None	46,XY,der(9)del(9)(p23)dup(9)(p23p12)	CMA (Oligo V6.2)	Loss (P)	arr cgh 9p23p24.3(RP11-19G1->RP11-165F24)X1, 9p13.1p23(CTD-2349C9->RP11-3L8)X3	9.7 Mb in 9p23p24.3 and 26 Mb in 9p13.1p23	ish der(9)(RP11-31F19-,RP11-381H12++,CTD-2349C9++)	66 deleted and 285 duplicated	De Novo
	#13	Gonadal Dysgenesis	None	46,XY,der(9)del(9)(p24pter)dup(9)(p23p12)	CMA (V.5.0)	Loss (P)	arr cgh 9p24.1-pter(GS-43N6-RP11-165F24->RP11-146B14)X1	6.7	n/a	58	De Novo
	#9	Swyer Syndrome	None	46,XY,del(9)(p23)	CMA (V.5.0)	Loss (P)	arr cgh 9p24.3 (RP11-165F24->RP11-31F19)X1, 7p22.3(RP11-90P13->RP1-164D18 16D1S)X3	0.26	n/a	4	Unavailable
	#14	Gonadal Dysgenesis	None	46,XY,var(Y)(q12)	CMA (Oligo V6.3)	Loss (P)	arr cgh 9p23(RP11-19G1)x1	0.20	ish del(9)(p23p23)(RP11-19G1dim)	1	Unavailable
**10q26**	#15	Ambiguous Genitalia (Referral)	Multiple Congenital Anomalies	XY t(12;15), del10q	CMA (V.5.0)	Loss (P)	arr cgh 10q26.1q26.3 (RP11-338O1->RP11-426G8)x1	57.4	n/a	620	Unavailable
**19q12**	#22	Ambiguous Genitalia (Referral)	None	46,XY	CMA (V.5.0)	Loss (P)	arr cgh 19q12q13.11(RP11-142B21->RP11-618P17)x1	5.7	Interstitial deletion between band 19q12 to 19q13.2 by partial karyotype	49	De Novo
**22q11.2**	#24	Ambiguous Genitalia (Referral)	Developmental Delay	46,XX,t(2;9)(p25.3;p22.1)	CMA (V.5.0)	Gain (P)	arr cgh 22q11.21(RP11-186O8->RP11-165F18)x3	1.6	ish dup(22)(q11.2q11.2)(RP11-165F18x3)	69	Unavailable
	#25	Smith-Lemli-Opitz Syndrome	None	n/a	CMA (V.5.0)	Loss (P)	arr cgh 22q11.21(RP11-186O8->RP11-165F18)x1	1.6	ish del(22)(q11.2q11.2)(F5-)	69	Unavailable
	#26	Ambiguous Genitalia (Referral)	Wilms Tumor Tetralogy of Fallot, Developmental Delay, Mental Retardation	46,XX,del(22)(q11.21q11.23)	CMA (V.5.0)	Loss (P)	arr cgh 22q11.2(RP11-186O8->RP11-165F18)x1	1.6	ish del(22)(q11.2q11.2)(RP11-165F18-)	69	Unavailable
**Yp11.31**	#27	Mixed Gonadal Dysgenesis	None	45,X[26]/46,X,idic(Y)(q11.2][24]	CMA (Oligo V6.2)	Loss (P)/Mosaic	Deletion in Yp11.31q11.22: Mos Turner arr cgh Y(RP11-112L19->RP11-223K9)x0	18.7	n/a	189	Unavailable
	#28	Mixed Gonadal Dysgenesis	None	n/a	CMA (Oligo V6.3)	Loss (P)/Mosaic	Deletion in Yp11.31q11.22: Mos Turner arr cgh Yp11.31q11.22(RP11-400O10->RP11-223K9)x1	18.8	Retrospective: nuc ish Xcen(DXZ1x1),Ycen(DYZ3x0)[88]/Xcen(DXZ1x1),Ycen(DYZ3x1)[112]	192	Unavailable
	#29	Ambiguous Genitalia (Referral)	None	46,X,add(X)(p22.3)	CMA (V.6.1)	Gain (P)/Mosaic	Presence of Yp11.31p11.2: arr cgh Yp11.31p11.2(RP11-112L19->RP11-418M8)x1	6 Mb of Yp on distal short arm of Chr. X	Retrospective: ish der(X)(CEP X+,LSI SRY+)	44	Unavailable
	#30	Gonadal Dysgenesis	None	n/a	CMA (V.6.1)	Gain (P)/Mosaic	Duplication in Yp11.31q11.21: arr cgh Yp11.31q11.21(RP11-112L19->RP11-460B21)x2	10.7	Retrospective: ish Yp11.31(SRY-)[5]/Yp11.31(SRY+)[3]/Yp11.31(SRY++)[12]	116	Unavailable

**Table 3 pone-0015392-t003:** List of pathogenic (P) copy number variations in patients presenting with cryptorchidism.

Locus	Case	GU defects	Associated anomalies	Concurrent G-banded karyotype	Array platform	CNV(Signifi-cance)	Rearrangements seen in CMA	Min. size (Mb)	Secondary FISH	Min. genes	Inheritance
**5p15.2**	#7	Cryptorchidism (inguinal)	None	46, XY	CMA (Oligo V6.3)	Gain (P)	arr cgh 5p15.2(RP11-327L20)x3	0.1	n/a, *	1-8	Present in maternal
**10p14**	#16	Cryptorchidism (abdominal)	VACTERL Syndrome, imperforate Anus, Unilateral Renal Agenesis	n/a	CMA (Oligo V6.3)	Gain (P)	arr cgh 10p14 (RP11-796C22)x3	0.064	FISH: ish dup(10)(p14)(RP11-590M7x3)	3	De Novo
**12q24**	#18	Cryptorchidism (inguinal)	Transposition of Great Vessels, Ventricular Septal Defect, Developmental Delay, Epilepsy, Hypotonia	46,XY,dup(12)(q24.2q24.31)	CMA (Oligo V6.3)	2 Gains (one UCS and one P)	arr cgh 12q24.13 (RP3-329E11, RP1-66E7)x3, 12q24.21q24.31(RP11-902D13-RP11-197N18)x3	2 gains (12q24.13>116Kb; 12q24.21-12q24.3>7.9Mb)	n/a	3 for 12q24.13 - 107 for 12q24.21q24.31	Unavailable
**15q11**	#19	Cryptorchidism	None	n/a	CMA (Oligo V6.2)	Loss (P)	arr cgh 15q11.2q12(RP11-289D12—>RP11-345N11)x1	3.9	n/a	126	Unavailable
**Xq28**	#33	Cryptorchidism (abdominal)	Congenital Diaphragmatic Hernia	46, XY	CMA (Oligo V6.3)	Gain (P)	arr cgh Xq28 (RP11-479B17)x3	0.159	ish dup(X)(q28)(RP11-479B17x3)	1	De Novo

**Table 4 pone-0015392-t004:** List of pathogenic (P) copy number variations in patients presenting with hypospadias.

Locus	Case	GU defects	Associated anomalies	Concurrent G-banded karyotype	Array platform	CNV(Signifi-cance)	Rearrangements seen in CMA	Min. size (Mb)	Secondary FISH	Min. genes	Inheritance
**2q22**	#2	Hypospadias, Cryptorchidism	None	46, XY	CMA (Oligo V6.5)	Loss (P)	arr cgh 2q22.2q22.3(RP11-734C21->RP11-294G19)x1	2	ish del(2)(q22.3q22.3)(RP11-249G19-)	9	Unavailable
**5p15**	#5	Hypospadias (midshaft)	None	46, XY	CMA (Oligo V6.3)	Gain (P)	arr cgh 5p15.31(RP11-46O23)x3	0.065	n/a, *	1	Present in maternal DNA
**12p13**	#17	Hypospadias	None	n/a	CMA (Oligo V6.2)	Loss (P)	arr cgh 12p13.31p13.2(RP11-69M1->RP11-656E20)x1	2.3	ish del(12)(p13.31p13.31)(RP11-69M1-)dn	65	De Novo
**16p11**	#20	Hypospadias	Cleft palate	46,XY	CMA (Oligo V6.5)	Loss (P)	arr cgh 16p11.2(29729970 - 29861142)x1	0.131	ish del(16)(p11.2p11.2)(RP11-301D18-)dn	10	De Novo
**Xq28**	#34	Hypospadias (penoscrotal)	None	46, XY	CMA (Oligo V6.3)	Gain (P)	arr cgh Xq28 (RP11-479B17)x3	0.159	n/a, *	1	Unavailable

**Table 5:** List of copy number variations of unclear clinical significance (UCS) in 46,XY DSD patients.

Locus	Case	GU defects	Associated anomalies	Concurrent G-banded karyotype	Array platform	CNV (Signifi-cance)	Rearrangements seen in CMA	Min. size (Mb)	Secondary FISH	Min. genes	Inheritance
**4q35**	#3	Hypospadias (Glanular)	None	46, XY	CMA (Oligo V6.3)	Gain (UCS)	arr cgh 4q35.2(RP11-354H17)x3	0.075	n/a, *	0	Present in maternal DNA
**5p15.2**	#7	Cryptorchidism (inguinal)	None	46, XY	CMA (Oligo V6.3)	Gain (UCS)	arr cgh 5p15.2(RP11-327L20)x3	0.1	n/a, *	1-8	Present in maternal
**7p22.1**	#8	Ambiguous Genitalia (Referral)	Mild Developmental Delay, Mental Retardation, Failure To Thrive	n/a	CMA (Oligo V6.3)	Loss (UCS)	arr cgh 7p22.1(RP11-160E17)x1	0.16	ish del(7)(p22.1p22.1)(RP11-160E17dim)	5	Present in maternal DNA
**7p22.3**	#9	Swyer Syndrome	None	46,XY,del(9)(p23)	CMA (V.5.0)	Gain (UCS)	arr cgh 7p22.3(RP11-90P13->RP1-164D18 16D1S)X3, 9p24.3 (RP11-165F24->RP11-31F19)X1	0.25	n/a, *	3	Unavailable
**8p23.2**	#11	Hypospadias	Developmental Delay, mental retardation	46,XY	CMA (V.6.1)	Gain (UCS)	arr cgh 8p23.2(RP11-82K8)x3	0.05 Kb	nuc ish dup(8)(p23.2)(RP11-82K8x3)	1	Unavailable
**8q12**	#10	Ambiguous Genitalia (Referral)	None	46, XY	CMA (V.5.0)	Loss (UCS)	arr cgh 8q12.2(RP11-414L17->RP11-174G1)x1	0.41	ish del(8)(q12.2q12.2)(RP11-33I11-)	4	Unavailable
**16p13**	#6	Ambiguous Genitalia (Referral)	None	46,XY,der(5)(qter->q34::p14->qter)	CMA (Oligo V6.2)	Loss (UCS)	arr cgh 16p13(RP11-349E19)x1, 5p15.33p14.2(RP11-487N22->RP11-91L13)x1	0.14	nuc ish dup(16)(q24.3) (RP11-566K11x3)	1	Unavailable
**16q24.3**	#21	Hypospadias (corona)	None	46,XY	CMA (Oligo V6.3)	Gain (UCS)	arr cgh 16q24.3 (RP11-566K11)x3	0.078	nuc ish dup(16)(q24.3) (RP11-566K11x3)	9	Not in maternal. Paternal unavailable
**Xq12**	#32	Cryptorchidism (inguinal)	Spina Bifida	46, XY	CMA (Oligo V6.3)	Gain (UCS)	arr cgh Xq12(RP11-349K4)x3	0.07	n/a, *	0	Present in maternal DNA

Regardless of the GU condition, the size of CMA detected anomalies ranged from 50 kilobases to 57.4 Mb with an average defect size of 5.5 Mb, which is at the limit of the resolution of routine karyotype (Tables 2–5). Importantly, none of the imbalances smaller than 5 Mb, which represent about 70% of the identified CMA defects, were detected by concurrent high-resolution karyotype analysis (Tables 2–5). For the cases of imbalances larger than 5 Mb, CMA analysis had proven to provide a better definition of the structural defect than the karyotype (patients 6, 9, 12, 13, 18- see Tables 2–5). Moreover, most of the detected imbalances (70.6%) were subtelomeric defects (Tables 2–5), which are known to be difficult to characterize by G-banding due to their location in the distal G-negative staining regions of the chromosomes. The most illustrative finding was seen in patient 15 with a referring diagnosis of ambiguous genitalia (Table 2). A large deletion of 57.4 Mb spanning the subtelomeric 10q26 was only suspected by karyotype, but was successfully detected by CMA analysis. Interestingly, this deletion encompassed *FGFR2*, a particularly noteworthy candidate gene in light of recent studies in rodents that found evidence for its role in testis formation and male sex determination [24], [25]. While gene variants of *FGFR2* may influence the risk of hypospadias in humans [26], conditional inactivation of *FGFR2* in mouse models resulted in blockade of the XY-specific gonad growth and disruption of testis differentiation, leading to a male-to-female sex reversal phenotype. The characterization of *FGFR2* as a sex-determining gene in the mouse suggests that the CMA detected human haploinsufficiency of *FGFR2* is a strong candidate defect underlying the phenotype of abnormal male genital development in patient 15.

Interestingly, in three unrelated CMA screened patients (27, 28 and 30; Table 2), a low-level mosaic state became apparent after CMA screening and retrospective analysis of the karyotype. Interphase FISH performed on blood smears in which multiple cell lineages coexist, was requested after CMA testing, to verify mosaicism in these children. The conventional karyotype was normal since it examined only the cell population of stimulated T lymphocytes. The fact that CMA analysis performed on DNA from uncultured blood cells was able to improve the detection of low level mosaicism missed by cytogenetic analysis, is of significant clinical importance, especially for the diagnosis of genital ambiguity.

### Strong Association of *De Novo* Copy Variants with Human Disorders of Sex Development

The inheritance of the FISH-confirmed CMA defects was investigated by CMA testing. Parental samples were not available for all patients, leading to an underestimation of the clinically significant abnormalities in the present evaluation. *De novo* occurrences were noted for: (i) deletions in 1p36.33, 9p23p24 and 19q12-q13.11 for probands presenting with a referring diagnosis of ambiguous genitalia; (ii) duplications in 10p14 and Xq28 for cryptorchid children; (iii) and deletions in 12p13.31-p13.2 and 16p11.2 for patients with hypospadias (Table 6). Importantly, these *de novo* copy number changes were found to be more frequent in patients with congenital genitourinary defects than in control individuals without GU abnormalities (28 out of 8951), with an association that is statistically significant (*P* = 6.08×10^−12^; Fisher's exact test) (Tables 6 and 7).

**Table 6:** *De novo* clinically relevant copy number changes detected in patients presenting with disorders of sex development (DSD).

ID	Locus	DSD diagnosis	CNV	Start Position	Size (Mb)	Genes	% In Non-GU	% In GU	*P* value	Karyotype	Inh
17	12p13.31p13.2	Hypospadias	Loss	7,987,984	2.306	65	0.01	1.11	1.9×10^−2^	46,XY	*dn*
20	16p11.2	Hypospadias	Loss	29,729,970	0.131	10	0.07	1.11	5.7×10^−2^	46,XY	*dn*
34	Xq28	Hypospadias	Gain	154,703,321	0.158	1	0	2.22	9.9×10^−3^	46,XY	*dn*
33	Xq28	Cryptorchidism	Gain	154,703,321	0.158	1	0	2.22	9.9×10^−3^	46,XY	*dn*
16	10p14	Cryptorchidism	Gain	12,011,806	0.064	3	0.02	1.11	2.9×10^−2^	n/a	*dn*
1	1p36.33	Ambiguous Genitalia	Loss	799,476	1.257	65	0.17	1.11	1.4×10^−1^	46,XX	*dn*
12	9p23p24.3	Ambiguous Genitalia (Gonadal dysgenesis)	Loss	356,238	9.774	66	0.04	2.22	1.4×10^−3^	46,XY,der(9)del(9)(p23)dup(9)(p23p12)	*dn*
13	9p24.1-pter	Ambiguous Genitalia (Gonadal dysgenesis)	Loss	1	6.785	58	0.04	2.22	1.4×10^−3^	46,XY,der(9)del(9)(p24pter)dup(9)(p23p12)	*dn*
9	9p24.3	Ambiguous Genitalia (Gonadal Dysgenesis)	Loss	356,238	0.259	4	0.04	2.22	1.4×10^−3^	46,XY,del(9)(p23)	*dn*
22	19q12q13.11	Ambiguous Genitalia	Loss	33,828,527	5.638	49	0	1.11	9.9×10^−3^	46,XY	*dn*

*Footnotes:*

Minimal size of the spontaneous aberrations (Mb) and the number of the encompassing HGNC (Hugo Gene Nomenclature Committee) genes (G) (NCBI Build v35.1) were indicated.

*P* values were based on two-tailed Fisher's exact test comparing the frequency of each spontaneous event in cases versus controls. Significance threshold was set at *P* = 5.0×10^−2^.

Abbreviations: Inh: Inheritance, *dn*: de novo.

**Table 7:** *De novo* CMA detected events are more enriched in GU patients than in individuals without urogenital abnormalities.

Sample Group	Total Patients*	*Patients with de novo* events	Ratio	*P* value
Genitourinary Defects	90	10	0.11	6.08×10^−12^
Non-Genitourinary Defects	8951	28	0.003	

*Footnotes*: Two-tailed Fisher's exact test was used to evaluate the association of CMA detected *de novo* events with urogenital defects. *: GU cases (n = 90 out of the total of 116 analyzed GU children) and non GU controls (n = 8951) run only on CMA V.6.1 and CMA Oligo V6, since *de novo* events were specifically observed in GU patients screened with these two qualitatively comparable platforms (n = 10; see Table 6); [21]. See Statistical Analysis in Methods for details.

To a lesser extent, imbalances inherited from a phenotypically normal maternal parent were also considered since the rearrangements may be causative without necessarily translating into similar abnormal GU traits in the female genitourinary tract. Thus, maternally inherited copy changes were considered as of unclear clinical significance and noted as: (i) deletion in 7p22.1 for ambiguous genitalia; (ii) duplications in 4q35.2 and 5p15.31 for hypospadias; (iii) duplication in 5p15.2 and in the androgen receptor insensitivity region, Xq12 for cryptorchidism (Table 5).

Hence, the present analysis shed light on spontaneous chromosomal rearrangements affecting novel and unsuspected gene-enriched regions that have potential to contribute to the pathogenesis of human genital development.

### Unrelated Patients Presenting with Similar Genital Traits Shared Common Affected Loci

**Figure 2:**
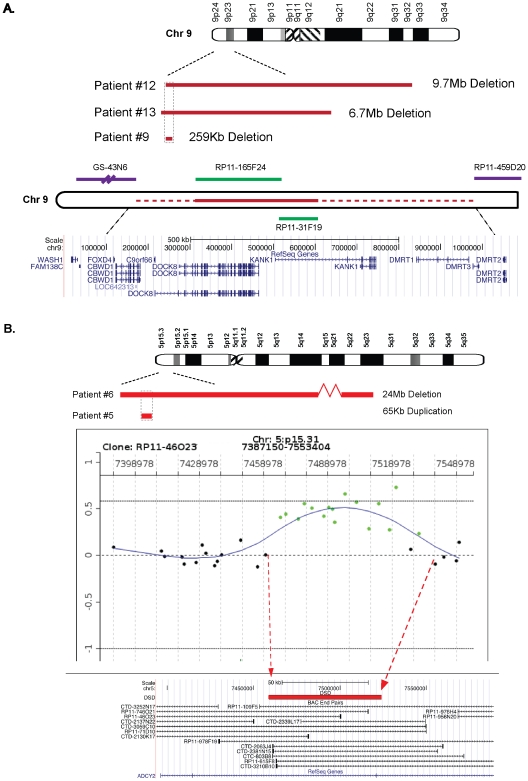
Overlapping Chromosomal Rearrangements in DSD patients. **A**. **Delineation of a minimal human 9p sex reversal deletion.** Schematic representation of the overlapping CMA detected 9p deletions in three unrelated 46,XY patients presenting with gonadal dysgenesis. A minimal common 260 Kb region was defined. Map showing the BAC clones covering the critical sex determination region and the normal flanking clones (RP11-459D20 and GS-43N6). A UCSC genome browser view (*May 2006* Human Assembly) of the *RefSeq* genes encompassing the minimal 9p24.3 sex-reversing region was presented. **B**. **Structural variation shared by unrelated patients presenting with distinct urogenital defects, may affect master regulator(s) of human genital development.** A common genomic interval of 65 Kb in the cytoband 5p15.31 was deleted in patient 6 with genital ambiguity and duplicated in patient 5 with hypospadias. CMA detection of the 65 Kb duplication in patient 5 and a UCSC genome browser view (*May 2006* Human Assembly) of the encompassed *ADCY2* gene were presented.

The causal link of the CMA defects to the GU phenotype was further strengthened by the fact that common overlapped loci were shared by unrelated probands having similar genital defects. Spontaneous deletion of the 9p23p24 region was found in patients 9, 12, and 13, all with gonadal dysgenesis (Table 2). A minimal common region of overlap included 260 kb of 9p24.3 (Figure 2A). This smallest reported sex-reversing 9p deletion appears therefore as a hotspot for the pathogenesis of sex determination. It encompasses *KANK1*, *DOCK8* and *DMRT* genes. The testis specific *DMRT1* is worthy of mention, since it encodes a protein-sharing domain homology with the doublesex (*Dsx*) of *Drosophila* and *Mab3* of *Caenorhabditis*. Both of these genes are crucial for the normal sexual development of these organisms. Genetic inactivation of *DMRT1* demonstrated its requirement for the development of the male gonad [27], but did not lead to sex reversal in XY mice, suggesting the involvement of additional interacting factors in order to phenocopy the human phenotype. One of such gene candidates could be *KANK1* since it is highly expressed in the mouse embryonic genital tract (http://www.genepaint.org) and is able to physically interact and regulate the subcellular localization of beta catenin whose activation in normal XY mice has been shown to disrupt the male program and result in male-to-female sex-reversal [28], [29].

In addition to the 9p region, the locus Yp11.31 was an expected recurrent hit in patients with ambiguous genitalia, since it encompasses *SRY*, the testis-determining gene (Table 2).

### Unique Similar Loci were Abnormal in Patients Presenting with Different Urogenital Traits

Identical unique loci were affected in patients presenting distinct genital phenotypic traits, which suggest that structural perturbations within these segments may alter master regulator(s) of multiple processes of human sexual development. Indeed, a large deletion of the subtelomeric cytoband 5p15 detected in the patient 6 with genital ambiguity overlapped a region duplicated in patient 5 with hypospadias (Figure 2B). The fact that the shared genomic interval spanned the locus of the Cri-du-Chat syndrome, which includes hypospadias among its various clinical features, provides support for a causative link of the 5p15 defect to the GU phenotype. The encompassed gene, *ADCY2*, appeared as an ideal candidate controlling genitourinary development since it presents a high and specific expression pattern in the testis as well as in the developing genital tract (http://www.genepaint.org). Moreover, the *ADCY2* encoding protein regulates the intracellular levels of cyclic AMP, a crucial second messenger in major regulatory pathways involved in the biogenesis of the genital system such as Sonic Hedgehog signaling.

The DiGeorge syndrome critical region 22q11.2 was also found duplicated in patients with ambiguous genitalia, while its deletion was seen in patients presenting with GU abnormalities in association with Smith-Lemli-Opitz syndrome or Wilms tumor (Table 2). This region may have a dosage sensitive gene(s) that plays a role in the development of the genitourinary system in humans.

In parallel, alterations occurring on 2q22 and Xq28 loci were found in patients with cryptochidism and hypospadias (Tables 3–4). These genomic regions may contain candidate genes that regulate a common protagonist(s) or pathway(s) controlling both testicular descent and formation of the male urethra. The deletion 2q22 in patient 2 presenting with cryptorchidism and hypospadias was in the region associated with Mowat-Wilson syndrome (MWS). Among anomalies frequently observed in MWS are urogenital anomalies including hypospadias and undescended testis. MWS is a genetic condition caused by heterozygous mutations or deletions of *ZEB2* (zinc finger E-box binding homeobox 2 gene), a protein that interacts with a receptor-mediated, activated full-length SMAD. *ZEB2* is strongly expressed in the developing murine genital tract (http://www.genepaint.org). Knockout mice models of *ZEB2* presented reproductive system defects ([30], http://www.informatics.jax.org). Moreover, *ZEB2* has been shown to modulate *Wnt* signaling, a critical pathway for the development of the genital tract [31]. Hence, *ZEB2* appears as a potential candidate involved in the male urogenital development.

Taken together, our findings highlight for the first time the presence of previously unrecognized chromosomal imbalances as potential genetic risks factors in disorders of sexual development and illustrate how a microarray-based technology provides a powerful alternative to traditional cytogenetic and gene-mapping approaches for discovering contributing factors in disease of complex etiology.

## Discussion

The development of male reproductive system is a complex process controlled by delicate networks that specify sex-specific differentiation, organogenesis and endocrine function. The fragility of these regulatory cascades is illustrated by the high prevalence of genitourinary defects in newborns. These inborn urogenital anomalies present difficult challenges for the parents and the physicians, as care of these children is complicated by surgical, psychological, social and sexual concerns. The gold standard for genetic diagnosis remains a karyotype analysis and an endocrine profile but findings in intersex cases are not always informative. Indeed, only a small portion of these developmental aberrations can be attributed to defects in the synthesis of testosterone or adrenal steroid hormones, receptor alterations, exogenous modifiers or obvious numerical and structural chromosomal alterations, such as Klinefelter syndrome. The underlying causes of the majority of “idiopathic” cases remain to be discovered. In this study, the use of a clinically validated microarray (CMA) revealed the existence of cryptic imbalances strongly associated with defects of urogenital development or recurrently found in patients with DSD. These chromosomal aberrations were mostly too small to be detected by the routinely ordered karyotype, which has a limited resolution of 5–10 Mb, depending on the quality of chromosome preparations. Many of these genomic anomalies went also largely undetected because they were located in subtelomeric loci, which are notoriously difficult to characterize by G-banding. Moreover, mild or isolated cases of hypospadias and cryptorchid patients are usually not referred for genetic testing, while this study proved that this subset of patients harbored structural variation that may convey defective urogenital traits.

Most of the detected chromosomal aberrations encompassed one to a few hundred genes including known gonad-determining genes (*SRY* and *DMRT1*) as well as novel candidate genes such as *FGFR2*, *KANK1*, *ADCY2* and *ZEB2*. Changes in dosage or structure of genes within the affected DNA segments might lead to haploinsufficiency or altered transcription profiles, which may disturb the intricate fine-tuned network of genes controlling the human genital development. Clinically relevant examples of gene dosage alterations have already been documented for factors controlling mammalian sex development. For instance, deletion of the sex-determining gene *WNT4* is responsible for the masculinization of XX mouse pups, while its duplication and overexpression in humans leads to XY sex reversal [32], [33]. Duplications of large segments of DNA containing *DAX1* or *SOX9* also cause sex reversal [34], [35]. Thus, our findings contribute in a coherent manner to strengthen the emerging concept that sex determination and differentiation are dosage sensitive at multiple steps of their pathways. In addition to dosage effects, imbalances may lead to disruption of regulatory sequences that control the expression of neighboring genes; thus, in some cases, a gene related to genital development may lie adjacent to the detected deletion or duplication. For instance, a submicroscopic 258 Kb deletion, detected 11,320 bp upstream of *DAX1* in a 21-year-old 46,XY female, may lead to a loss of regulatory sequences and position-effect upregulation of *DAX1* expression [36].

Our findings provide support for the genomic basis of human disorders of sexual development and call for genome-wide CNV screenings which may, due to their extended coverage, reveal a higher proportion of germline mutations associated with urogenital defects. Enrichment in candidate genes for human sexual development is subsequently bound to increase. Our present study using the clinically established CMA platform was motivated by a rapid translation of our findings to the clinical arena. Molecular testing, such as with CMA, could significantly impact patient care by assisting the pediatric urologists and neonatologists in diagnosis. Genetic counseling offered to families based on the identification of pathogenic rearrangements may provide parents with essential clinical information pertaining to the child's diagnosis and permit proper estimates of the risk of recurrence for subsequent pregnancies. *De novo* imbalances are expected to have a very low risk of recurrence but it may be useful in future pregnancies to check for gonadal mosaicism in the parents. An unbalanced translocation identified by CMA may reveal a balanced translocation in a carrier parent and thus chances for a chromosomally abnormal future pregnancy would be as high as 25%. In vitro fertilization and pre-implantation genetic diagnosis could provide these couples with a possible alternative path to parenthood, specifically in case of severe genital ambiguity.

In conclusion, this study presented structural DNA variation as a potential underlying etiology for human disorders of sexual development. Frequent disease-causing submicroscopic gains and losses of DNA segments were detected across the genome and strongly associated with defective urogenital traits. This has been achieved with significantly higher resolution and greater clinical yield than standard routine karyotype, thus making this array-based CGH screen as a genetic test of choice in diagnosis. While GU defects cases arise among newborns without clear etiology, this study offers novel loci to dissect for determining key genes involved in the human sexual development.
